# Digital health literacy, social support, and online health information-seeking behavior profiles among nursing students: a latent profile and structural equation modeling analysis

**DOI:** 10.3389/fpubh.2026.1939476

**Published:** 2026-09-10

**Authors:** Lijie Yang, Liujiang Liang, Huihui Hu, Kaili Pan, Qingqing Ding, Xiaohong Meng

**Affiliations:** 1 Guangxi Medical University Cancer Hospital, Nanning, China; 2 School of Nursing, Guangxi Medical University, Nanning, China

**Keywords:** digital health literacy, latent profile analysis, nursing students, online health information-seeking behavior, social support, structural equation modeling

## Abstract

**Background:**

Online health information-seeking behavior (OHISB) is important for nursing students, yet its heterogeneity and associations with digital health literacy and social support remain unclear.

**Objective:**

To identify OHISB profiles among nursing students and examine the hypothesized indirect association between digital health literacy and profile membership through perceived social support.

**Methods:**

This cross-sectional study included 772 nursing students. Data were collected using a sociodemographic questionnaire, the eHealth Literacy Scale, the Online Health Information-Seeking Behavior Scale, and the Perceived Social Support Scale. Latent profile analysis identified OHISB profiles. Multinomial logistic regression examined predictors of profile membership, with three-step analyses accounting for classification uncertainty. Structural equation modeling assessed indirect associations through perceived social support using bootstrap confidence intervals.

**Results:**

Among the nursing students, latent profile analysis identified three distinct online health information-seeking behavior profiles: high (*n* = 286, 37%), moderate (*n* = 328, 42.5%), and low (*n* = 158, 20.5%) OHISB profiles. Place of origin, family structure, household income, education level, grade, and social support were associated with profile membership (*p* < 0.05). In adjusted three-step analyses, higher perceived social support was associated with lower odds of high- (OR = 0.956) and moderate-OHISB (OR = 0.959) membership relative to low-OHISB membership. Digital health literacy was positively associated with perceived social support (*B* = 0.293, *p* < 0.001). Perceived social support was negatively associated with high- (*B* = −0.311, OR = 0.733, *p* < 0.001) and moderate-OHISB membership (*B* = −0.215, OR = 0.807, *p* < 0.001) relative to low-OHISB membership. Direct effects of digital health literacy were not significant (*p* = 0.637 and 0.511). Significant indirect effects were observed for high versus low (*a* × *b* = −0.091, 95% bootstrap CI: −0.136 to −0.060) and moderate versus low (*a* × *b* = −0.063, 95% bootstrap CI: −0.102 to −0.036) contrasts.

**Conclusion:**

Online health information-seeking behavior among nursing students was heterogeneous and characterized by three profiles. Perceived social support showed significant indirect associations between digital health literacy and OHISB profile membership. These findings highlight the potential importance of social support, while the cross-sectional design precludes causal inference.

## 1 Introduction

The rapid growth of digital healthcare has revolutionized the dissemination and accessibility of health information. An individual’s capacity to effectively utilize digital tools to locate, interpret, and apply health information, known as digital health literacy, has become a pivotal determinant of personal health outcomes. Online health information-seeking behavior (OHISB), defined as the active pursuit, evaluation, and selection of health-related information online to satisfy knowledge needs and reduce uncertainty about one’s health status (1), is integral to evidence-based nursing practice and directly influences patient care quality (2). This competency is especially critical for nursing students, who must make informed decisions about their own health and accurately communicate health information to patients, thereby shaping health behaviors.

The Extended Model of Health Information Seeking Behavior conceptualizes individuals as both active seekers and passive recipients of information, shaped by environmental and personal factors (3). Empirical studies reveal significant associations among social support, digital health literacy, which encompasses the skills to search for, understand, critically evaluate, and integrate electronic health information from online sources to make informed health decisions, and OHISB (4–6). The relationship between digital health literacy and OHISB is complex and varies across populations (7, 8). Theoretically, digital health literacy may precede perceived social support because greater ability to access, evaluate, and use digital health resources may facilitate engagement with online health communities and interpersonal resources, thereby expanding individuals’ perceived access to social support. Social support may serve as a critical mediator, modulating the translation of digital health literacy into effective OHISB. However, the association may also be mutually beneficial, as greater social support may provide informational and interpersonal resources that help individuals improve their digital health literacy.

Research on OHISB has predominantly focused on college students (9, 10), older adults (11, 12), and patients with chronic conditions (13), while studies specifically targeting nursing students remain limited. Moreover, prevailing variable-centered approaches often overlook heterogeneity in individuals’ OHISB behaviors. Latent profile analysis (LPA), a person-centered statistical method, addresses this gap by identifying distinct OHISB subgroups based on response patterns, enabling nuanced characterization of group-specific traits (14).

This study employs LPA to delineate OHISB profiles among nursing students and use structural equation modeling (SEM) analysis to examines how digital health literacy influences perceived social support, which in turn affects the OHISB profiles. The findings aim to inform tailored, tiered health education interventions. We hypothesize that:

*H1*: Nursing students can be classified into multiple distinct OHISB profiles.

*H2*: Perceived social support is hypothesized to mediate the association between digital health literacy and OHISB profiles.

*H3*: The indirect association between digital health literacy and OHISB profile membership through perceived social support differs across OHISB profile contrasts.

## 2 Materials and methods

### 2.1 Study design and participants

A cross-sectional survey was conducted among nursing students from three medical colleges in Guangxi, China, between January and February 2026. Participants were recruited via a WeChat-based online questionnaire using convenience sampling. To prevent duplicate submissions, each IP address was restricted to a single response.

Nursing students currently enrolled in undergraduate or junior college programs, regardless of academic year, were eligible. Students with severe mental illness that could impede questionnaire completion were excluded. Consistent with *a priori* data-quality criteria, responses completed in fewer than 90 s or showing straightlining (identical responses across all items) were excluded. Finally no questionnaires were excluded by these criteria (minimum completion time = 101 s).

Sample size adequacy was evaluated in relation to the complexity of the planned analyses, including a three-indicator, three-class LPA, multinomial regression of profile membership, and a single-mediator SEM with a three-category latent outcome. Previous simulation studies have shown that LPA performance depends on sample size, the number and quality of indicators, the number of latent classes, and the separation between classes, rather than on a single fixed minimum sample size. In the present study, the three latent profiles comprised 286 (37.0%), 328 (42.5%), and 158 (20.5%) participants, respectively, with the smallest class containing 158 participants. The selected three-class solution also demonstrated good classification quality (entropy = 0.865). Thus, the final sample of 772 provided adequate representation of each latent class and sufficient observations for the subsequent multinomial regression and mediation analyses.

The study was approved by the institutional review board of the Science and Technology Ethics Committee of Guangxi Medical University Cancer Hospital (approval no. KY2026788), and informed consent was obtained from all participants.

### 2.2 Measures

#### 2.2.1 Demographic characteristics

A self-designed questionnaire was used to collect demographic characteristics, including age, sex, student origin (urban/rural), family structure (nuclear/extended), family monthly income (<3,000, 3,000–5,999, and >6,000 CNY), education level (junior college/undergraduate), academic year (freshman to senior), and self-rated health (good/fair/poor).

#### 2.2.2 Online health-seeking behavior scale (OHSB)

The OHISB was assessed using the scale, which comprises three subscales: information seeking (8 items), offline supplementation or replacement of professional care (11 items), and online social support seeking (18 items) (15). Items are rated on a 5-point Likert scale (5 = very frequent, 4 = frequent, 3 = sometimes, 2 = occasionally, and 1 = never), yielding a total score of 37–185, with higher scores indicating greater engagement in OHISB. The Cronbach’s α coefficient of the scale was 0.981, indicating very high internal consistency and substantial homogeneity among the items. Subscale sum scores were used as indicators in the LPA. A confirmatory factor analysis (CFA) supported the three-factor structure (RMSEA = 0.055, CFI = 0.893, SRMR = 0.056), justifying the use of the three subscale scores.

#### 2.2.3 Digital health literacy

The eHealth Literacy Scale (eHEALS) was used to assess digital health literacy. It was originally developed by Norman et al. (7) and translated and validated in Chinese by Guo et al. (16). It consists of eight items, and items are rated on a 5-point Likert scale ranging from 1 (“strongly disagree”) to 5 (“strongly agree”). The total score ranges from 8 to 40, with higher scores indicating greater digital health literacy. The Cronbach’s α coefficient of the scale was 0.972. CFA supported a unidimensional structure (after allowing three residual correlations between conceptually similar items: CFI = 0.943, TLI = 0.906, SRMR = 0.041, RMSEA = 0.087), with all standardized loadings ≥ 0.58. Measurement invariance of the eHEALS across sex was supported (ΔCFI = 0.008 < 0.01 for configural vs. metric models, with invariant intercepts).

#### 2.2.4 Perceived social support

Perceived social support was measured using the 12-item Perceived Social Support Scale (PSSS), originally developed by Zimet et al. (17) and translated and validated in Chinese by Jiang et al. (18). It comprises three dimensions: family support, friend support, and other support, with a total of 12 items. Items are rated on a 7-point Likert scale ranging from 1 (“strongly disagree”) to 7 (“strongly agree”). The total score ranges from 12 to 84, with higher scores reflecting greater perceived social support. The Cronbach’s α coefficient of the scale was 0.922. CFA supported the three-factor structure (RMSEA = 0.034, CFI = 0.987, SRMR = 0.021).

### 2.3 Statistical analysis

#### 2.3.1 Descriptive statistics

Descriptive statistics were used to summarize participant characteristics by OHISB profile. Categorical variables were presented as frequencies and percentages, and continuous variables were summarized as means and standard deviations, as appropriate. Group differences in categorical variables were assessed using *χ*^2^ tests, whereas differences in digital health literacy and perceived social support across latent profiles were examined using the BCH three-step method to account for classification error.

#### 2.3.2 Latent profile analysis

The LPA was conducted on the three OHISB subscale sum scores using Mplus 8.3. Models with 1–5 classes were estimated and compared on the AIC, BIC, sample-size-adjusted BIC, entropy, the Lo–Mendell–Rubin likelihood ratio test (LMR), and the parametric bootstrap likelihood ratio test (BLRT). The final model was selected based on statistical criteria, interpretability, and class sizes. Sensitivity analyses included a four-class solution and a re-analysis using mean subscale scores (subscale sums divided by item counts) as indicators, which reproduced the three-class solution exactly (*n* = 286, 158, 328; entropy = 0.865).

#### 2.3.3 Three-step regression

To examine sociodemographic factors and social support as predictors of profile membership while accounting for classification uncertainty, a multinomial logistic regression was estimated using the manual three-step approach, with the low-OHISB profile as the reference. Results are reported as unstandardized logit coefficients (B), standard errors, Wald *χ*^2^, odds ratios (OR) with 95% confidence intervals (CI), and *p*-values.

#### 2.3.4 Mediation analysis (SEM)

A structural equation model tested whether perceived social support mediated the association between digital health literacy and OHISB profile membership. Digital health literacy predicted social support (path a); social support predicted profile membership (paths b₁, b₂, multinomial logit); and digital health literacy directly predicted profile membership (paths c′₁, c′₂). Profiles were modeled as a latent categorical variable (three classes) to avoid treating class membership as observed. The model was estimated with the ML estimator and ALGORITHM = INTEGRATION, using a multinomial logit link for the categorical outcome. Indirect effects (a × b) and their 95% CIs were computed via MODEL CONSTRAINT and 1,000 bootstrap draws. A sensitivity analysis re-estimated the model with perceived social support as a latent variable indicated by its three subscales.

Analyses were conducted in Mplus 8.3; two-tailed *p* < 0.05 was considered statistically significant.

## 3 Results

### 3.1 Profile differences in demographic characteristics, digital health literacy, and social support

Participants’ ages ranged from 18 to 37 years, with a mean of (20.75 ± 1.79) years.

The three profiles differed significantly in all demographic variables (all *P* ≤ 0.05). Digital health literacy and perceived social support showed an inverse gradient across profiles: both were highest in the low-OHISB profile (32.12 ± 5.94 and 67.75 ± 11.48, respectively) and lowest in the high-OHISB profile (30.36 ± 5.73 and 58.62 ± 10.89, respectively), with the moderate-OHISB profile showing intermediate values (31.23 ± 5.55 and 62.37 ± 11.53, respectively). BCH-adjusted comparisons confirmed significant differences across profiles in digital health literacy (*p* = 0.008) and perceived social support (*p* < 0.001) (Table 1).

**Table 1:** Univariate analysis of factors influencing latent profiles of OHISB among nursing students.

Variable	Total (*n* = 772)	Low OHISB (*n* = 158)	Moderate (*n* = 328)	High (*n* = 286)	*χ* ^2^	*P*
Sex					37.45	<0.001
Male	126 (16.3)	51 (32.3)	43 (13.1)	32 (11.2)		
Female	646 (83.7)	107 (67.7)	285 (86.9)	254 (88.8)		
Student origin					36.51	<0.001
Urban	261 (33.8)	42 (26.6)	150 (45.7)	69 (24.1)		
Rural	511 (66.2)	116 (73.4)	178 (54.3)	217 (75.9)		
Family structure					75.71	<0.001
Nuclear	196 (25.4)	80 (50.6)	78 (23.8)	38 (13.3)		
Extended	576 (74.6)	78 (49.4)	250 (76.2)	248 (86.7)		
Family income (CNY)					83.41	<0.001
<3,000	128 (16.6)	14 (8.9)	40 (12.2)	74 (25.9)		
3,000–5,999	265 (34.3)	30 (19.0)	109 (33.2)	126 (44.1)		
>6,000	379 (49.1)	114 (72.2)	179 (54.6)	86 (30.1)		
Education					81.87	<0.001
Junior college	194 (25.1)	17 (10.8)	53 (16.2)	124 (43.4)		
Undergraduate	578 (74.9)	141 (89.2)	275 (83.8)	162 (56.6)		
Grade					60.11	<0.001
Freshman	104 (13.5)	49 (31.0)	31 (9.5)	24 (8.4)		
Sophomore	304 (39.4)	58 (36.7)	123 (37.5)	123 (43.0)		
Junior	217 (28.1)	34 (21.5)	108 (32.9)	75 (26.2)		
Senior	147 (19.0)	17 (10.8)	66 (20.1)	64 (22.4)		
Self-rated health					14.30	0.006
Good	425 (55.1)	101 (63.9)	183 (55.8)	141 (49.3)		
Fair	313 (40.5)	56 (35.4)	131 (39.9)	126 (44.1)		
Poor	34 (4.4)	1 (0.6)	14 (4.3)	19 (6.6)		
Digital health literacy	31.09 ± 5.73	32.12 ± 5.94	31.23 ± 5.55	30.36 ± 5.73	–	0.008^†^
Social support	62.08 ± 11.75	67.75 ± 11.48	62.37 ± 11.53	58.62 ± 10.89	–	<0.001^†^

Data are *n* (%) or mean ± SD.

^†^BCH-adjusted test across profiles (37), accounting for classification error.

### 3.2 Identification of OHISB profiles

**Figure 1:**
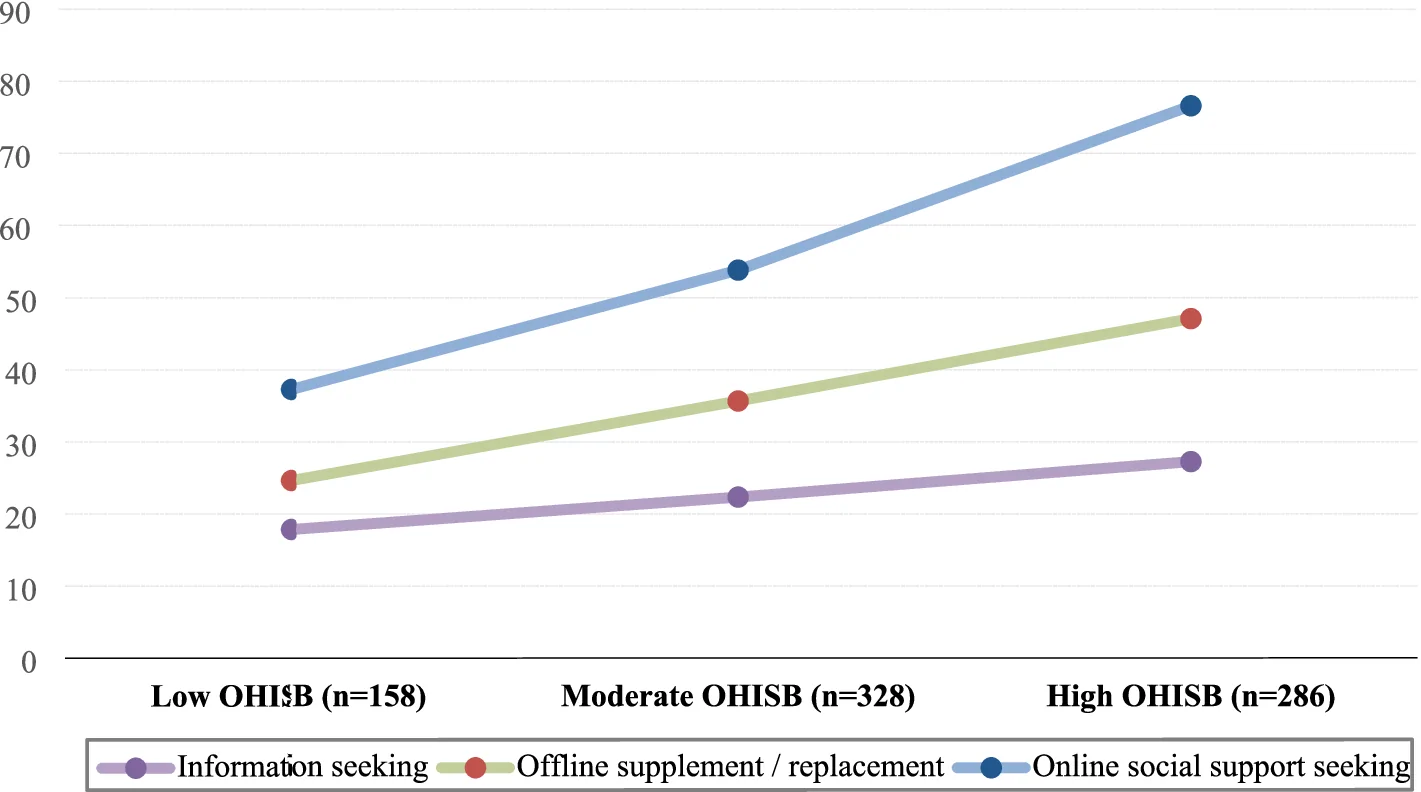
Nursing students’ online health-seeking behavior profiles. The three profiles were characterized by low, moderate, and high scores across the three OHISB subscales: Information seeking (8 items, range 8–40), offline supplementation/replacement of professional care (11 items, range 11–55), and online social support seeking (18 items, range 18–90).

Latent profile analysis on the three OHISB subscale sum scores identified a three-profile solution as the most parsimonious and interpretable model (Table 2). Although information criteria (AIC, BIC, aBIC) continued to decrease with additional classes, the LMR test was non-significant for four- and five-class solutions (*p* = 0.058 and 0.059, respectively), and the five-class solution contained a very small class (*n* = 6, 0.8%) lacking practical meaning. The three-class solution showed the highest entropy (0.865), all average latent class probabilities exceeded 0.90 (range 0.929–0.957), and a sensitivity analysis using mean subscale scores reproduced the three-class solution exactly (*n* = 286, 158, and 328; entropy = 0.865). The three-profile solution and the corresponding profile characteristics are presented in Figure 1.

**Table 2:** Model fit indices for the LPA solutions (*k* = 1–5).

Model	Log-likelihood	Free params	AIC	BIC	aBIC	Entropy	LMR (*P*)	BLRT (*P*)	Class sizes, *n* (%)
1-class	−8580.546	6	17173.09	17200.99	17181.93	–	–	–	772 (100.0)
2-class	−8162.656	10	16345.31	16391.80	16360.05	0.852	<0.001	<0.001	445 (57.6), 327 (42.4)
3-class	**−8007.463**	**14**	**16042.93**	**16108.01**	**16063.56**	**0.865**	**<0.001**	**<0.001**	**286 (37.0), 158 (20.5), 328 (42.5)**
4-class	−7963.627	18	15963.25	16046.94	15989.78	0.843	0.058	<0.001	159 (20.6), 218 (28.2), 315 (40.8), 80 (10.4)
5-class	−7925.548	22	15895.10	15997.37	15927.51	0.866	0.059	<0.001	6 (0.8), 307 (39.8), 159 (20.6), 219 (28.4), 81 (10.5)

Bold indicates the chosen model. LMR = Lo–Mendell–Rubin likelihood ratio test; BLRT = parametric bootstrap likelihood ratio test.

The three profiles were ordered by their OHISB subscale levels (Information Seeking, Offline Supplementation or Replacement of Professional Care and Online Social Support Seeking): 27.2/47.1/76.6 for the high-OHISB profile, 22.3/35.6/53.8 for the moderate-OHISB profile, and 17.9/24.7/37.3 for the low-OHISB profile, respectively. They were therefore labeled high-OHISB (*n* = 286, 37.0%), moderate-OHISB (*n* = 328, 42.5%), and low-OHISB (*n* = 158, 20.5%) profiles.

### 3.3 Multivariate analysis of factors influencing OHISB profiles among nursing students

Table 3 presents the three-step multinomial logistic regression with the low-OHISB profile as the reference. Perceived social support was negatively associated with profile membership: each one-point increase in social support was associated with lower odds of membership in the high-OHISB (OR = 0.956, 95% CI 0.925–0.988, *p* = 0.010) and moderate-OHISB (OR = 0.959, 95% CI 0.927–0.991, *p* = 0.015) profiles relative to the low-OHISB profile. Rural student origin was associated with lower odds of high-OHISB membership (OR = 0.404, *p* = 0.004), whereas extended family structure (OR = 3.051, *p* < 0.001), lower family income (<3,000 CNY: OR = 2.727, *p* = 0.022), junior college education (OR = 4.470, *p* < 0.001), and higher academic year (sophomore to senior: ORs 2.864–7.123, all *p* < 0.01) were associated with higher odds of high-OHISB membership.

**Table 3:** Multivariate analysis of factors influencing OHISB profiles among nursing students (*n* = 772).

Variable	*B*	SE	Wald *χ*^2^	OR (95% CI)	*P*
High vs. Low OHISB					
Social support (per 1 point)	−0.045	0.017	6.61	0.956 (0.925–0.988)	0.010
Female (ref: Male)	0.491	0.345	2.03	1.634 (0.83–3.21)	0.154
Rural (ref: Urban)	−0.906	0.318	8.15	0.404 (0.22–0.75)	0.004
Extended family (ref: Nuclear)	1.115	0.311	12.90	3.051 (1.66–5.61)	<0.001
Income <3,000 (ref: >6,000)	1.003	0.439	5.24	2.727 (1.15–6.44)	0.022
Income 3,000–5,999	0.942	0.332	8.04	2.564 (1.34–4.92)	0.005
Junior college (ref: Undergraduate)	1.497	0.399	14.12	4.470 (2.04–9.77)	<0.001
Sophomore (ref: Freshman)	1.052	0.376	7.81	2.864 (1.37–5.98)	0.005
Junior	1.343	0.437	9.46	3.832 (1.63–9.02)	0.002
Senior	1.963	0.504	15.20	7.123 (2.65–19.1)	<0.001
Not-good health (ref: Good)	0.069	0.269	0.07	1.071 (0.63–1.81)	0.798
Moderate vs. Low OHISB					
Social support (per 1 point)	−0.042	0.017	5.94	0.959 (0.927–0.991)	0.015
Female (ref: Male)	0.525	0.330	2.52	1.690 (0.89–3.23)	0.112
Rural (ref: Urban)	−1.484	0.293	25.66	0.227 (0.13–0.40)	<0.001
Extended family (ref: Nuclear)	0.961	0.278	11.94	2.614 (1.52–4.51)	0.001
Income <3,000 (ref: >6,000)	0.285	0.467	0.37	1.330 (0.53–3.32)	0.541
Income 3,000–5,999	0.588	0.323	3.32	1.800 (0.96–3.39)	0.069
Junior college (ref: Undergraduate)	0.303	0.425	0.51	1.354 (0.59–3.11)	0.475
Sophomore (ref: Freshman)	0.861	0.369	5.45	2.366 (1.15–4.88)	0.020
Junior	1.208	0.402	9.03	3.347 (1.52–7.36)	0.003
Senior	1.598	0.466	11.74	4.942 (1.98–12.3)	0.001
Not-good health (ref: Good)	0.031	0.269	0.01	1.031 (0.61–1.75)	0.909

### 3.4 Mediating effect of social support on the relationship between digital health literacy and *OHISB*

**Figure 2:**
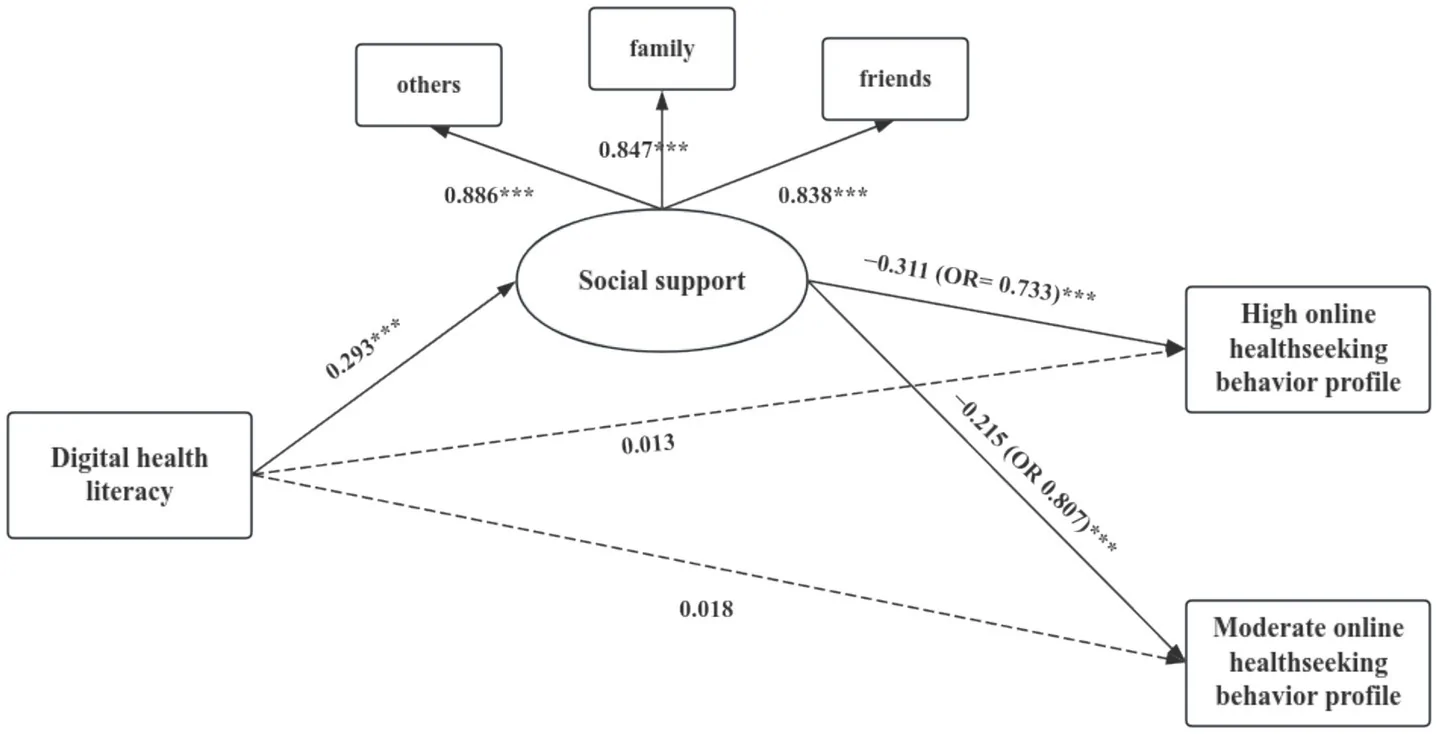
Path diagram of the hypothesized mediation model with perceived social support as a latent variable. ^***^ < 0.001.

Table 4 and Figure 2 summarize the mediation model. Digital health literacy was positively associated with perceived social support (*B* = 0.293, *p* < 0.001; path a). Perceived social support was negatively associated with membership in the high-OHISB (*B* = −0.311, OR = 0.733, *p* < 0.001) and moderate-OHISB (*B* = −0.215, OR = 0.807, *p* < 0.001) profiles relative to the low-OHISB profile (paths b₁, b₂). The indirect effects of digital health literacy on profile membership through social support were significant and negative for both the high-vs.-low (a × b = −0.091, 95% bootstrap CI − 0.136 ~ −0.060) and moderate-vs.-low (a × b = −0.063, 95% bootstrap CI − 0.102 ~ −0.036) contrasts, whereas the direct effects of literacy were non-significant (*p* = 0.637 and 0.511). A sensitivity analysis treating social support as an observed total score yielded consistent results (indirect effects −0.078 and −0.054).

**Table 4:** Mediation of the association between digital health literacy and OHISB profile membership through perceived social support.

Path	*B* (SE)	*P*	OR (95% CI)	Bootstrap 95% CI
a: Digital health literacy → Social support	0.293 (0.028)	<0.001	–	–
b₁: Social support → High vs. Low OHISB	−0.311 (0.061)	<0.001	0.733 (0.650–0.826)	–
b₂: Social support → Moderate vs. Low	−0.215 (0.056)	<0.001	0.807 (0.723–0.900)	–
c′₁: Literacy → High vs. Low (direct)	0.013 (0.028)	0.637	1.013	–
c′₂: Literacy → Moderate vs. Low (direct)	0.018 (0.028)	0.511	1.018	–
Indirect 1: Literacy → Support → High vs. Low (a × b₁)	−0.091 (0.019)	<0.001	–	−0.136 ~ −0.060
Indirect 2: Literacy → Support → Moderate vs. Low (a × b₂)	−0.063 (0.017)	<0.001	–	−0.102 ~ −0.036

B = unstandardized coefficient (logit scale for profile paths); OR = odds ratio; CI = confidence interval. Indirect effects = a × b, computed via MODEL CONSTRAINT; bootstrap CIs exclude zero, indicating significant indirect effects.

## 4 Discussion

### 4.1 Main findings and heterogeneity of OHIS

This study identified three distinct latent profiles of OHISB among nursing students: low (158 students, 20.5%), moderate (328 students, 42.5%), and high (286 students, 37.0%) profiles. This finding supports the first hypothesis and indicates substantial heterogeneity in OHISB among nursing students. The three profiles were characterized by relatively low, moderate, and high levels across the three dimensions of OHISB, including information seeking, offline supplementation or replacement of professional care, and online social support seeking. This finding is consistent with previous research suggesting that OHISB is multidimensional and shaped by multiple individual and contextual factors (8).

The observed heterogeneity may be explained by differences in individual characteristics, access to digital resources, and perceptions of online health information. Previous studies have shown that demographic characteristics, education level, and digital health literacy are associated with individuals’ ability and willingness to seek health information online (19–21). In addition, trust in online health information may influence how individuals search for and use digital health resources (22). Even among nursing students with professional training, difficulties in evaluating the credibility of online information may lead to inappropriate trust in low-quality sources or underuse of reliable information (23, 24).The findings show that students in the high-OHISB profile had the lowest levels of digital health literacy and perceived social support. However, students in the low-OHISB profile had the highest levels of both. This finding suggests that a higher frequency or intensity of OHISB should not necessarily be interpreted as more adaptive or higher-quality health information behavior. The OHISB scale includes not only general information seeking but also offline supplementation or replacement of professional care and online social support seeking. Thus, high scores may be related to intensive reliance on online information or online support rather than effective use of health information. Previous research has linked lower eHealth literacy with problematic online health behaviors, including cyberchondria and health anxiety, supporting the possibility that intensive online health information use may sometimes reflect uncertainty or dependence rather than greater competence (25).

Multinomial regression further showed that several sociodemographic characteristics were associated with profile membership. Rural origin was associated with lower odds of high- and moderate-OHISB membership, while extended family structure, lower household income, junior college education, and higher academic year were associated with higher odds of high-OHISB membership. These findings suggest that the patterns of online health information use may be related to socioeconomic resources and digital access. Previous research has emphasized the digital divide and sociodemographic characteristics can influence digital health literacy and Internet-based health information use (26–29). The finding that lower educational attainment was associated with higher odds of high-OHISB membership also suggests that intensive online health information seeking does not necessarily indicate greater digital competence.

Therefore, nursing education should focus not only on encouraging online health information seeking but also on strengthening students’ ability to critically evaluate and appropriately use digital health information.

### 4.2 Sociodemographic factors and profile differences

Multinomial logistic regression showed that rural student origin, extended family structure, lower family income, junior college education, and higher academic year were independently associated with OHISB profile membership. Specifically, rural origin was associated with lower odds of high- and moderate-OHISB membership, while extended family structure, lower household income, junior college education, and higher academic year were associated with higher odds of high-OHISB membership.

The observed rural–urban difference is consistent with research highlighting the impact of the digital divide and socioeconomic conditions on access to and use of digital resources (27, 30). Educational attainment and academic progression may also influence digital skills and online information-search strategies. However, the finding that junior college students had higher odds of high-OHISB membership suggests that intensive online health information seeking does not necessarily correspond to greater digital health literacy (21, 31). The person-centered studies among nursing students have similarly shown the differences in digital literacy profiles and associations with educational characteristic (32, 33).

Family structure was also associated with OHISB profiles, with students from extended families were more likely associated with moderate- and high-OHISB membership. This association showed differences in family communication, resource sharing, or access to interpersonal information sources.

These findings suggest that interventions should be tailored according to students’ educational and socioeconomic backgrounds rather than assuming that greater online information-seeking intensity represents greater competence. We can conduct more structured training in source evaluation, verification, and critical appraisal of online health information, which may really benefit students with high OHISB but relatively low digital health literacy.

### 4.3 Association between digital health literacy, social support, and OHISB

This study found a positive association between digital health literacy and perceived social support (*B* = 0.293). Digital health literacy was related to perceived social support, because people with greater ability to use digital health resources may take a more active part in online health communities and interpersonal resources, thereby getting more social support. This interpretation is consistent with previous research linking digital/eHealth literacy with the effective use of digital health resources and interpersonal support (34, 35).

However, the mediation analysis does not establish a causal sequence. Perceived social support was negatively associated with membership in the high-OHISB (*B* = −0.311, OR = 0.733) and moderate-OHISB (*B* = −0.215, OR = 0.807) profiles relative to the low-OHISB profile. The indirect effects of digital health literacy through perceived social support were significant and negative for both the high-vs.-low (a × b = −0.091, 95% bootstrap CI − 0.136 ~ −0.060) and moderate-vs.-low (a × b = −0.063, 95% bootstrap CI − 0.102 ~ −0.036) contrasts. Higher digital health literacy was statistically associated with higher perceived social support, which was in turn associated with low OHISB profiles. Several explanations are possible. First, differences in the operationalization of OHISB may be important: the present scale includes behaviors involving supplementation or replacement of professional care and online social support seeking. Higher scores may be associated with seeking a lot and even being dependent. Second, students with lower offline social support may compensate by seeking information and validation online. Third, demographic and socioeconomic factors may shape both digital competence and online information-use patterns. Therefore, high-frequency online health information seeking should not automatically be equated with high-quality or adaptive information behavior.

The absence of a statistically significant direct effect of digital health literacy should also not be interpreted as proof of “full mediation.” Rather, the results indicate a statistically significant indirect association through perceived social support, while the direct associations were not statistically significant. Given the cross-sectional design, these findings should be interpreted as an indirect association consistent with the proposed mediation model rather than evidence of a causal mechanism.

### 4.4 Directionality, alternative models, and implications

In this study, we placed digital health literacy before perceived social support in the model because students who are better at finding and evaluating online health information may also be better able to search and use support resources, whether online health communities or face-to-face networks. The positive association we observed (*B* = 0.293) is consistent with this idea. The reverse direction is just as plausible, however. Strong social networks may themselves build digital skills—through encouragement, informal guidance, and everyday opportunities to practice using health information online (36). With cross-sectional data we cannot tell which pathway comes first, and the two may well reinforce each other. We therefore regard the mediation model as one reasonable specification rather than evidence of temporal order, and there is no causal claim.

The practical implications follow from the profile pattern regardless of direction. Students in the high-OHISB profile sought the most health information online while reporting the lowest digital health literacy and perceived social support. For these students, it’s not useful to continuously seek online. We should provide more training in critical appraisal on judging sources, verifying claims, and recognizing unreliable content. In addition, real-world support from peers, faculty, and family could also help students get more information, instead of only depending on online health information seeking. We offer these suggestions cautiously, because a cross-sectional study cannot establish that improving literacy or support would actually reduce heavy online health information use.

## 5 Limitations

This study has several limitations. First, its cross-sectional design restricts the ability to establish causal or temporal relationships among variables; therefore, future research should employ longitudinal designs to validate these findings. Although digital health literacy was theoretically specified as an antecedent of perceived social support, the cross-sectional design cannot establish temporal ordering, and reciprocal or alternative pathways remain possible; therefore, the indirect effects should be interpreted as hypothesized associative relationships rather than causal mechanisms. Second, the sample was drawn exclusively from nursing colleges within a single region, which limits the generalizability of the results; thus, multi-center and cross-regional studies are necessary to enhance representativeness and applicability. Because institution identifiers were not collected, clustering by institution could not be explicitly modeled, and residual between-institution heterogeneity may have affected the precision of the estimates. Third, although LPA statistically identifies distinct classes, the labeling and interpretation of these categories remain somewhat subjective. Future research should incorporate qualitative methods to gain deeper insights into the underlying behavioral motivations and contextual factors influencing these profiles. The LPA was based on three OHISB subscale scores rather than item-level indicators; although CFA and sensitivity analyses supported the identified structure, alternative profile solutions cannot be completely excluded. Three-step and BCH-adjusted analyses were used to account for classification uncertainty, although some uncertainty in profile assignment may remain. Fourth, all variables were measured using self-reported questionnaires, which may introduce common method bias despite the use of validated instruments. Recall and social desirability biases may also have affected the responses; future studies should incorporate objective behavioral measures where possible.

## 6 Conclusion

This study classified nursing students’ OHISB into low, moderate, and high profiles. The three profiles were characterized as low (20.5%), moderate (42.5%), and high (37.0%) OHISB profiles. Student origin, family structure, income, education level, grade, and social support were significant predictors of these OHISB profiles. Higher perceived social support was associated with low OHISB profiles. Digital health literacy was positively associated with perceived social support, while higher perceived social support was negatively associated with membership in the high OHISB profiles. Mediation analysis indicated significant indirect associations of digital health literacy with OHISB profile membership through perceived social support. These findings highlight social support as a key bridge linking digital health literacy to OHISB. However, given the cross-sectional design, these findings should be interpreted as associative rather than causal, and the directionality of the digital health literacy–social support relationship remains uncertain. Interventions should focus on enhancing both digital skills and social support networks, adopting stratified, dynamic, and personalized educational strategies. Particular attention may be warranted for students with intensive online health-information seeking but lower digital health literacy and perceived social support.

## Data Availability

The raw data supporting the conclusions of this article will be made available by the authors, without undue reservation.
